# Epidemiology and Control of Rabies in Cattle and Equines in Rondônia State, a Brazilian’s Legal Amazon Area

**DOI:** 10.3390/ani13182974

**Published:** 2023-09-20

**Authors:** Débora Naihane Alves Sodré, Gabriel Augusto Marques Rossi, Luis Antonio Mathias, Marco Antonio de Andrade Belo

**Affiliations:** 1 IDARON—Agency of Agrosilvopastoral Sanitary Defense of Rondônia State, Cacoal 76964-062, RO, Brazil; 2 Department of Veterinary Medicine, Brazil University (UB), Descalvado 13690-000, SP, Brazil; 3 Department of Veterinary Medicine, Vila Velha University (UVV), Vila Velha 29102-920, ES, Brazil; 4 Department of Pathology, Reproduction and One Health, São Paulo State University (UNESP), Jaboticabal 14884-900, SP, Brazil

**Keywords:** bats, *Desmodus rotundus*, *Lyssavirus*, viruses, zoonosis

## Abstract

**Simple Summary:**

Rabies is a fatal zoonotic disease and a significant cause of economic losses in cattle and equine farms located in South America. In this continent, it is primarily transmitted to these hosts through bites of the hematophagous bat *Desmodus rotundus*. This survey investigated the presence of rabies in cattle and equines in the state of Rondônia, situated in the Brazilian’s Legal Amazon region, spanning from 2002 to 2021. The study correlated these findings with the preventive measures implemented by the local health agency (IDARON) for rabies management. Over this timeframe, there were 201 reported cases in cattle and 23 in horses. Both herbivores exhibited a declining trend in rabies incidences. No specific within-year patterns in rabies occurrence was identified, and there were variations in epidemic periods. The reduction in rabies incidence risk among cattle and horses was attributed to the ratio of treated bats and the sale of vaccines in the area while, on the other hand, the number of educational printed materials contributed to the increasing the incidence. Despite the diminishing trend of rabies in this Amazon Rainforest region, which can be attributed to the efforts of the animal health agency, the disease remains endemic, necessitating ongoing surveillance and prophylactic strategies for its control.

**Abstract:**

Rabies is a fatal neglected tropical zoonosis, and its significance for domestic herbivores in the rural cycle is probably associated with rainforest deforestation, livestock, and agricultural expansion. This epidemiological survey aimed to study the occurrence of rabies in bovines and equines in the state of Rondônia, located in the Brazilian’s Legal Amazon, between the years 2002 and 2021, correlating these findings with the prophylactic strategies adopted by the local sanitary agency for rabies control. During this period, 201 cases were observed in bovines and 23 in equines. A downward trend in rabies incidence was observed for both domestic herbivores. Rabies did not show a higher occurrence in any specific time of the year, and epidemic periods varied during some years for bovines and equines. Using the Generalized estimating equations (GEE) method, a multiple model approach was obtained with the explanatory variables significantly associated with the decrease in rabies incidence in cattle and horses during the study period: the ratio of treated bats and ratio of vaccine doses sold. Furthermore, the ratio of printed educative material was positively associated with rabies incidence. Despite a decreasing trend in rabies occurrences in this Amazon rainforest area, likely due to the actions taken by the animal sanitary agency, rabies remains endemic and requires monitoring, as well as prophylactic strategies to control this disease.

## 1. Introduction

Rabies is transmissible to all mammals and is caused by the neurotropic rabies virus (RABV) of the genus *Lyssavirus*. This zoonotic disease has a fatality rate of almost 100.00% in humans and animals and nowadays remains a global threat, resulting in around 59,000 human deaths per year [1]. Annually, the occurrence of rabies results in a global estimated cost of USD 8.6 billion [2]. Canines are considered by the World Health Organization as the most important source of infection for the zoonotic transmission of rabies worldwide, but the transmission through the bites of vampire bats or contact with saliva from infected herbivores on open cuts or wounds also occurs in countries such as those placed in South America [2].

In the last decade in Brazil, rabies has been transmitted to humans via bites by hematophagous bats (*Desmodus (D.) rotundus*) (52.60%), dogs (23.70%), non-human primates (10.50%), and felines (10.50%) [3], and there has been a growing number of reports regarding *D. rotundus* bites on humans, posing a potential threat to public health within this country [4]. *D. rotundus* are also the main transmitters of rabies to domestic herbivores in Brazil (100.00%, *n* = 50 [5] and 99.20%, *n* = 650 [6]), causing a high caseload of vampire-bat variant rabies in areas of Brazil where geographic conditions support a high number of bat vampire shelters [7,8]. Rabies occurrence in livestock animals is frequently reported, with cattle being the most affected among the livestock species [9]. This disease is considered an important cause of economic loss for cattle farmers [10]. Additionally, the importance of this disease in equines has recently been on the rise in Brazil [11].

For the control of rabies in cattle and equines in Brazil, the following control strategies are recommended by the federal government: the monitoring and control of hematophagous bats through the application of anticoagulant poison, mandatory vaccination of bovines and equines in focus and peripheral high-risk areas, voluntary vaccination of these animals in low-risk areas, mandatory notification of suspected cases of rabies or vampire-bat shelters detection, investigation of suspected cases, conducting laboratory tests for disease diagnosis, and implementing animal and human health education programs [12]. Anticoagulant poisons (Warfarin paste) are fatal when ingested by bats. Population control using anticoagulant poison occurs via two methods: they are either applied topically to bats to be transmitted through allogrooming, or they are administered to livestock for subsequent ingestion by bats during blood feeding. After applying the poison to the animals individually, it is expected that the population control within the shelters will be achieved. However, the efficacy of culling bats for rabies management continues to be a contentious topic of debate in the scientific community due to risks to wildlife [13].

Oliveira and colleagues [11] emphasized the need for attention to be directed specifically to the state of Rondônia, where this study was performed, due to the potential risk of rabies spread to transboundary areas. The monitoring of bat lyssaviruses in this area has also been recommended recently [14] and there is a need of adopting more effective prophylactic measures to control rabies in this area of the Brazilian’s Legal Amazon [9]. The animal health agency responsible for rabies control in domestic herbivores in the state of Rondônia, Brazil, is known as the “*Agência de Defesa Sanitária Agrosilvopastoril do Estado de Rondônia*” (IDARON), which translates to the Agency of Agrosilvopastoral Sanitary Defense of Rondônia State. IDARON operates in compliance with Ordinance No. 168, issued on 27 September 2005, by the Brazilian Ministry of Agriculture, Livestock, and Supply Chain (MAPA). This ordinance approves the Technical Manual for Herbivore Rabies Control, which guides IDARON’s actions [12].

Considering these factors, this study aimed to describe the epidemiology of rabies in bovines and equines in the state of Rondônia, Brazil, from 2002 to 2021, and correlate these findings with the prophylactic strategies implemented by the health agency for rabies control (IDARON) in livestock animals.

## 2. Materials and Methods

### 2.1. Study Design

**Figure 1:**
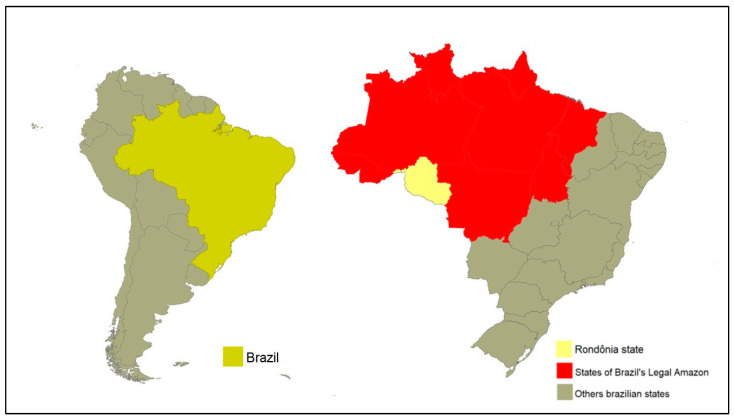
The maps illustrate Brazil’s location in South America and its composition of 26 states and a federal district. The country boasts an expansive area of the Legal Amazon, which encompasses not only the state of Rondônia, but also eight other states. Maps were created using the Terraview Software, version 4.2.0.

This study was conducted in Rondônia, a state located in the North Region of Brazil. Rondônia encompasses areas within the Brazilian’s Legal Amazon (Figure 1).

Spanning an area of 237,765,347 km², Rondônia consists of 52 municipalities. It is bordered by the states of Amazonas and Acre to the north, Mato Grosso to the east and southeast, and Bolivia to the southeast and west. The estimated population of Rondônia is around 1.7 million inhabitants, with 26.77% residing in rural areas, surpassing the national average of 15.64% [15].

This study encompassed all documented cases of rabies in bovines and equines in the specified area from 2002 to 2021. The data used in this study were directly obtained from the official records of the animal health agency responsible for this state, known as the “*Agência de Defesa Sanitária Agrosilvopastoril do Estado de Rondônia*” (IDARON). The annual reports (not published), compiled by the State Program of Herbivore Rabies Control of IDARON, are composed by the monthly data summaries obtained from the 52 sanitary units across all 52 municipalities of Rondônia. Regrettably, the number of cattle and equines tested for rabies diagnosis were not obtained during the data collection process. Consequently, we are unable to present the results regarding the proportion of infected animals among the total tested population. Nevertheless, all cases of rabies diagnosed in cattle and equines have been included in this study. The diagnosis tests for rabies were Direct Immunofluorescence, virus isolation in cell culture, or biological tests conducted on mice as recommended by the Technical Manual for Herbivore Rabies Control [12]. The animals considered as rabies cases were those that tested positive in any of these diagnostic tests [12]. The characterization of the variant associated with cases in the affected animals was not included in this study, and it was assumed that all cases were infected with *D. rotundus* bat-variant rabies virus due to its high frequency in livestock rabies in this country (>99.00% of cases) [5,6].

Data on various actions were collected from the reports provided by IDARON (not published). These parameters include the monthly numbers of: monitored bat shelters, identified bat shelters, bat capture events, captured vampire bats, hematophagous bats treated with anticoagulant poison, bats sent to the laboratory for rabies diagnosis, rabies tests diagnosis for rabies in animals (bovine, equines, and bats), cattle and horses diagnosed with rabies, number of vaccine doses sold for cattle and equines, doses of anticoagulant poison sold for farms, farms that purchased anticoagulant poison, meetings, courses and lectures related to health education of farmers, media disclosures (i.e., interviews and media disclosures in newspapers, radio, and television), and units of printed educational materials distributed [12].

In brief, the identification of shelters involves the farmer notifying IDARON in the event of occurrence or suspicion of rabies cases, as well as the presence of animals attacked by hematophagous bats or the existence of shelters of such species. When a shelter is identified on a farm it is registered, and these registered shelter records are monitored by IDARON at least once a year. During this monitoring, the bat population of that shelter is estimated, and the occurrence of attacks on the farm animals is checked. Additionally, a health education effort is carried out with the farm residents. In shelters frequented by hematophagous bats, whenever possible, specimens should be collected for submission to the laboratory. Moreover, if there is suspicion of rabies infection, specimens must be collected and sent to the laboratory for diagnosis. Capture events and bat treatment with anticoagulant poison can occur whenever there is a bat attack on farms and the farmer notifies IDARON. Not all shelters can be identified, so capture and treatment with anticoagulant poison can also take place in other types of facilities such as corrals, pigsties, and chicken coops. Additionally, bats can be collected when found dead or sick during monitoring or any other justified situation, such as during capture events [12].

### 2.2. Data Analyses

To organize, separate, and categorize the information, a database was created using the Excel 2010 program. The confidence interval for the incidence values was calculated using the Wilson method, employing the ‘binom’ package in R, enabling the comparison of incidence rates between cattle and horses. Additionally, this assessment was also conducted using the Fisher’s exact test [16].

The trend analysis was conducted using the ‘year of study’ as the independent variable, spanning from 2002 to 2021, and the ‘number of rabies cases’ in cattle and horses as the dependent variable. The open access Joinpoint Regression Program version 4.7.0.0 (US National Cancer Institute, Bethesda, MD, USA) (http://surveillance.cancer.gov/joinpoint/, accessed on 13 March 2023) and the Excel application were utilized for the time trend analysis.

For the trend analysis, Prais–Winsten regression was employed. The calculations were performed using the ‘Prais’ package [17] in the R^®^ software. Based on the regression coefficient and its standard error, the annual variation rate was calculated along with its corresponding 95.00% confidence interval [18].

To analyze for within year patterns of rabies occurrence, data on total cases per month, monthly incidence rate in cattle, and monthly incidence rate in equids were utilized. Monthly incidence rates were calculated by estimating the population for each month based on the annual population data gathered from IDARON using the arithmetic method [19]. Analysis for within year patterns was conducted using the Friedman test [16] in the Agricolae package of the R statistical software.

The potential association of the outcome variable, namely the incidence risk for bovine and equines (annual number of rabies positive/estimated population (millions)), was investigated with several possible explanatory variables. We calculated ratios between the annual number of sanitary actions and the number of farms. For each ratio, the unit (10×) was chosen to obtain a value not far from one digit. The explanatory variables included were:

ratio of monitored shelters = (annual number of monitored shelters/number of farms) × 10,000;

ratio of registered shelters = (annual number of registered shelters/number of farms) × 100,000;

ratio of capture events = (annual number of capture events/number of farms) × 10,000;

ratio of captured bats = (annual number of captured bats/number of farms) × 10,000;

ratio of treated bats = (annual number of treated bats/number of farms) × 10,000;

ratio of bats sent to laboratory = (annual number of bats sent to laboratory/number of farms) × 100,000;

ratio of diagnostic tests carried out = (annual number of diagnostic tests/number of farms) × 10,000;

ratio of vaccine doses sold = (annual doses of rabies vaccine sold/number of farms);

ratio of doses of anticoagulant poison sold = (annual doses sold/number of farms) × 1000;

proportion of farms that acquired anticoagulant poison or vaccines = (annual number of farms that acquired anticoagulant poison or vaccines/number of farms) × 100;

ratio of education actions = (annual number of meetings, courses and speeches/number of farms) × 1000;

ratio of media = (annual number of medias/number of farms) × 1000;

ratio of educative material = (annual number of educative material printed/number of farms) × 10.

The normality of the data for each variable was examined using the Shapiro–Wilk test [16], using the base R package. As not all variables showed a normal distribution, the association between individual explanatory variables and incidence risk for bovine and equines (millions) was investigated using the Spearman correlation test [16]. Multiple linear regression analysis was also done, but the results are not reported because the data did not fulfil the assumptions for valid use of this type of analysis because the residuals were not normally distributed and there were too many outliers among the residuals. Thus, the generalized estimating equations (GEE) method [20] from the “geepack” package in R was used to obtain a model with explanatory variables significantly associated with rabies incidence risk given the correlation among some variables. Explanatory variables with a *p*-value below 0.10 in the correlation test or in the simple GEE model were tested using simple GEE models. A multiple GEE model was constructed initially with all explanatory variables with *p* < 0.05 in the simple model. After that, a series of models were constructed by removing the one variable that had the highest *p* value > 0.05. Ultimately, this created a model that contained only variables with a significant (*p* < 0.05) association with rabies incidence. The estimate from the model indicated the direction and extent of the association.

## 3. Results

The number of rabies cases was higher in bovines; however, the incidence in equids was significantly (*p* = 3.981^−13^) higher (Table 1). There were cases of rabies every year only for cattle, whereas for horses it was observed non-annually.

**Figure 2:**
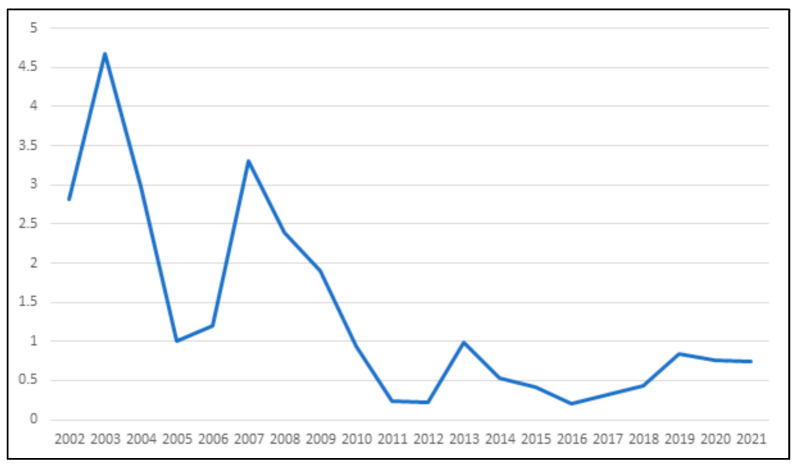
Total of positive animals (cattle and equines) divided by the number of farms (×10,000) for rabies occurrence in the state of Rondônia, from 2002 to 2021.

The number of infected animals (cattle and equines) were divided by the number of farms (×10,000) and this index is depicted in Figure 2.

Table 2 displays the results derived from the implementation of Prais-Winsten regression, which was utilized to assess the long-term trend of rabies in this state.

**Figure 3:**
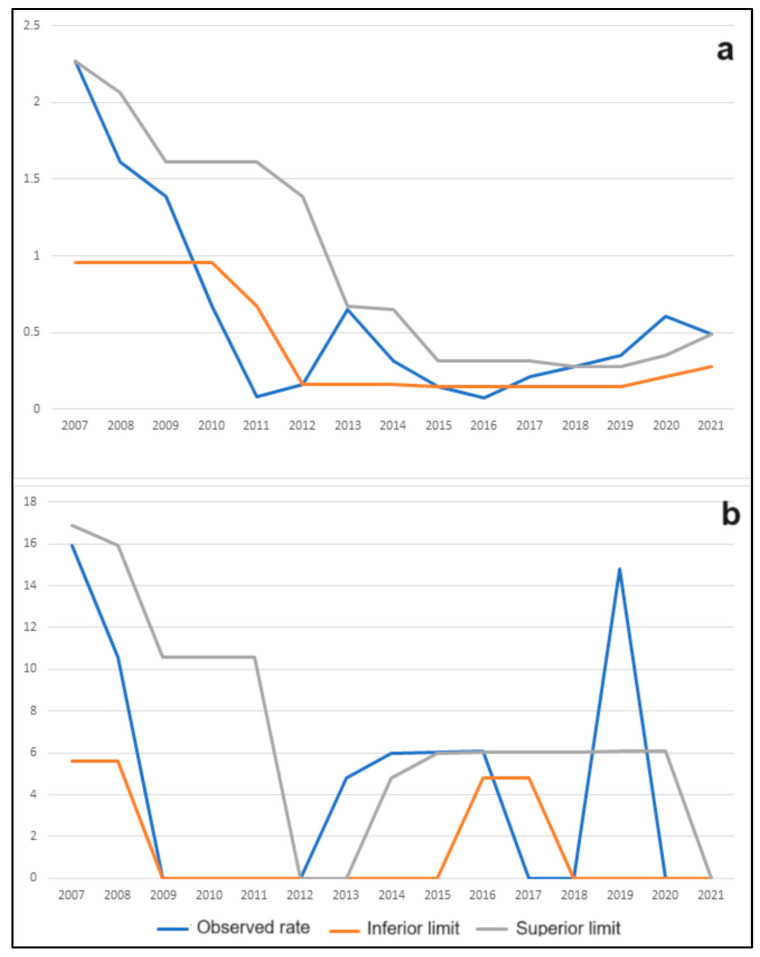
(**a**) The control diagram displays the lower limit of the expected range of occurrence (orange line), the upper limit of the expected range (gray line), and the observed incidence rate per one million cattle (blue line) from 2007 to 2021 in the state of Rondônia, Brazil; (**b**) The same control diagram is presented for equines over the same period.

The evaluation of within-year patterns in this disease was conducted, revealing no significant difference in rabies incidence among bovines (*p* = 0.2389) or equines (*p* = 0.7147) throughout the year. Additionally, the endemicity of rabies cases in bovines and equines was assessed. Figure 3 illustrates the epidemic periods from 2007 to 2021 for rabies in bovines (2019 and 2020) and equines (2013, 2014, and 2019).

The raw data pertaining to variables related to bat shelters, vaccine and anticoagulant poison commercialization, and educational initiatives can be found in Table 3, Table 4 and Table 5, respectively.

A summary of explanatory variables (ratios) can be found in Table 6.

Significant associations (*p* < 0.05) were observed by the Spearman correlation test between the outcome variable “incidence risk for bovines and equines” and the explanatory variables: ratio of monitored shelters (*p* = 0.000195), ratio of capture events (*p* = 0.01945), ratio of captured bats (*p* = 0.01784), and ratio of treated bats (*p* = 0.01133). All other variables were not significant (*p* > 0.05).

Using the GEE method, a multiple model was obtained with three explanatory variables significantly associated with the incidence risk for bovine and equines: ratio of treated bats, ratio of vaccine doses sold, and ratio of printed educative material (*p* < 0.05) (Table 7).

## 4. Discussion

Controlling livestock in rabies was associated with a substantial decrease in rabies cases between 2002 and 2021. This effort resulted in a sustained four-fold reduction in rabies incidence among herbivores, measured per million animals, by the year 2011.

The available evidence strongly indicates a global trend of under-reporting in rabies cases [2]. It is therefore plausible that under-reporting might also be present in the dataset used for this study. However, it is important to note that efforts have been made to mitigate this issue. The compulsory reporting of animals suspected to be rabies-infected to the local health agency (IDARON) has been in place [12], contributing to minimizing the potential for under-reporting. It is noteworthy that all instances of rabies cases in cattle and equine from the comprehensive IDARON database were meticulously incorporated into our study. Considering these measures, we maintain a high level of confidence in the representation of the actual number of cases within our dataset. However, we cannot ensure that they are not underestimated due to the potential occurrence of suspected cases not reported by farmers or veterinaries to IDARON.

Rabies affecting domestic herbivores is frequently reported in various regions of Brazil, including, at least, the states of Paraná [21], Mato Grosso [22,23], Rio Grande do Sul [24], and states located in the North Region of Brazil [8,25,26], where the state of Rondônia is located. The considerable majority (>99%) of rabies cases in herbivores attributed to the *D. rotundus*-variant rabies virus in other parts of Brazil [5,6] provides a solid rationale for investigating the potential connection between vampire-specific control strategies and the prevalence of rabies in cattle and equines examined in this study. The implemented control measures, which encompass efforts to manage *D. rotundus* populations, have the potential to substantially diminish the risk of rabies incidence in the local cattle and equine populations within this region (Table 1 and Table 7, Figure 2). In Brazil, human rabies also is predominantly transmitted via *D. rotundus* bites, as the transmission through dogs has been significantly reduced in this country [2]. Therefore, perhaps the control of rabies in hematophagous bats, focusing on the transmission of rabies to domestic herbivores, may also contribute to the prevention of rabies in humans.

In Rondônia, the incidence of rabies in equids was considered high (Table 1). Oliveira and colleagues [11] found a high incidence of equine rabies in Brazil (ranging from 2.8 to 137 per 100,000 horses), including an incidence in Rondonia (640 cases per 1 million equines) prior to our study period (2010–2019), which is considerably higher than that found in our study (127 per million). This difference may have occurred probably because we included a larger historical dataset. Furthermore, in our study, the incidence of rabies was significantly higher in equines. The impact of a relatively low number of cases in horses is more relevant within the horse population compared to the effect of bovine rabies on the cattle population. This is due to the considerably smaller size of the horse population in comparison to the cattle population, coupled with the fact that the number of cases in horses is also lower than that seen in cattle. It is worth highlighting that despite the difference in rabies incidences between cattle and horses in this study, prophylactic measures for this disease should be directed towards both species. This is because both species hold equal significance in the disease cycle and are managed through the same control measures.

Consistent with multiple other studies in Brazil [26,27,28,29,30,31,32], our analysis found no statistically significant within-year pattern of domestic herbivore rabies. The climatic characteristics of the North region, which lacks a rigorous winter and experiences high temperatures throughout the year, may contribute to the absence of distinct within-year patterns in bat populations and consequently minimize the patterns of rabies occurrence [5]. Our results demonstrate that control measures for rabies, including the annual vaccination of cattle and horses, should provide an effective reduction in the risk of rabies infection throughout the year.

The GEE model identified three control activities (negative associations: treating bats using anticoagulant poison and using vaccines in cattle and equines; positive association: printing educative materials) that were significantly associated (*p* < 0.05) with the rabies incidence during the study period. Our results are consistent with those of one study conducted in Brazil, specifically in the state of São Paulo, using official data from the local health agency for rabies control in domestic herbivores [31]. This study considered that controlling *D. rotundus* and vaccinating herbivores are the strategies responsible for the reduction in the cases of rabies in production animals. Another study conducted in the state of Minas Gerais, Brazil, highlighted the decrease in official bovine rabies tests between 1998 and 2006, as well as a decline in clinical cases of rabies. These authors attributed these improvements to enhanced control measures, including rabies vaccinations and the treatment of vampire bats using anticoagulant poison [32]. Our results corroborate what was reported by these authors, as we observed that the vaccination and application of anticoagulant poison were effective actions in reducing the occurrence of rabies in domestic herbivores.

A significantly more pronounced effect when employing anticoagulant poison for treating bats, in comparison to the impact of vaccinating cattle and equines, was observed. Employing anticoagulant poison to treat *D. rotundus* proves to be a potent control method. This approach targets multiple bats, thereby diminishing the potential for rabies transmission through the reduced interaction between all mammal populations, including herbivores and humans, and these bats. Consequently, the advantages of bat treatment likely extend beyond the realm of reducing rabies solely in herbivores and is likely to contribute to a decline in non-urban human rabies cases. In contrast, the vaccination’s impact is confined to disease prevention in the vaccinated individual, offering no direct benefit to human health.

Out of the six variables related to managing vampire bat populations, only one—treating bats with anticoagulant poison—showed a significant association with reducing the rabies incidence risk. Concerning shelter monitoring, capture events, and the number of captured bats, none of these factors displayed additional significance in the model. This was primarily because the datasets were intertwined and exhibited strong correlations. Treated bats constitute a subset of captured bats (rho = 0.99). Moreover, the numbers of captured bats (rho = 0.73) and capture events (rho = 0.79) correlate with the quantity of monitored shelters. Additionally, the rates of captured bats represent a subset of the capture events (rho = 0.91). While the figures related to shelter monitoring, capture events, and captured bats demonstrated high correlations (*p* < 0.1) in the Spearman correlation analysis, indicating a reduction in rabies cases, these associations were largely attributed to the impact of bat treatment alone. The capacity of the Generalized Estimating Equation (GEE) analysis to consider autocorrelation in the data allowed it to discern which specific bat shelter-based activity impacted rabies incidence. Despite not individually yielding significant reductions in rabies, the other shelter-based activities should remain integral to the control program, as they are essential for the effective treatment of bats.

Interestingly, while the application of anticoagulant poison to bats yielded significant results, the direct sale of these pastes to farmers did not exhibit a connection to reducing the incidence of rabies in cattle and equines. However, the values obtained for the number of farms that acquired anticoagulant poison were mixed with the acquisition of vaccines, which may have influenced our results (a bias). Despite both methods sharing the common approach of targeting bat populations in shelters through allogrooming behavior, the key distinction lies in the fact that one is directly applied to bats, whereas the other is administered to animals for ingestion by bats. Notably, the act of directly applying the poison to bats is executed by IDARON itself. Conversely, when it comes to the vampire bat pastes intended for application to animals, these are retailed in stores and then utilized by farmers to apply in domestic herbivores. Due to the unique nature of these actions, no correlation exists between them, elucidating why one strategy proved efficacious while the other did not. Possible reasons for this difference may include the sold poison not being used by farms, poison not being applied correctly, bats not coming into contact with poison, or the poison only affecting the contact bats (or fewer bats than when applied to captured bats).

The occurrence of rabies outbreaks in cattle is influenced by the remarkable adaptability of the bat species *D. rotundus* to artificial shelters and their proximity to the primary food source, which happens to be cattle in farms [33]. When cattle are granted free-range grazing access within expansive management systems encompassing forested areas, riparian zones, caves, secondary vegetation, as well as fragments and edges of vegetation, the ensuing conflict between vampire bats and cattle escalates in both magnitude and severity [34]. This scenario is widely presented in the state of Rondônia. Therefore, the control of hematophagous bats carried out by IDARON through anticoagulant poison contributed to the prevention of cases in livestock animals and possibly in humans, given that most human cases in Brazil are caused by attacks from hematophagous bats [3].

Furthermore, despite our data demonstrating the importance of controlling the populations of hematophagous bats for the transmission of rabies to domestic herbivores, the real effectiveness of these actions is currently questioned among the scientific community in a more general sense, as they may result in ecological impacts and risks for wildlife animals [13,34,35,36,37]. Bats are important in the Amazon ecosystem and the excessive population control may result in local or regional extinctions and unintended ecological consequences, such as killing other carnivores that ingest dead bats or the contamination of non-hematophagous bats with anticoagulant poison in multispecies colonies [37]. A promising alternative control involves vaccinating vampire bat populations using an effective transmissible rabies vaccine carried by a betaherpesvirus vector [38]. This strategy offers benefits compared to lethal treatments for bats; however, it is not currently accessible. The vaccination of bats would effectively address the ethical debates surrounding bat extermination and the potential extinction risk of a protected species within complex ecosystems, such as the Amazon Rainforest.

Our results also demonstrated the importance of vaccination for the control of rabies in production animals, which was associated with its incidence reduction. Mello and colleagues [9] reported 52 outbreaks of bovine rabies in 23 out of the 79 municipalities comprising the state of Mato Grosso do Sul, Brazil, spanning from 2010 to 2016. These authors concluded that the vaccination of cattle, regardless of herd size, was economically feasible and was recommended to minimize losses due to bovine rabies. Upon comprehensive analysis, the economic losses resulting from the documented outbreaks remained below the threshold of USD 5000.00 per farm. In a few outbreaks, losses ranged from USD 15,000.00 to USD 25,000.00. Among the 52 surveyed farms, the collective expense of vaccination would amount to less than USD 200.00 for 45 of them. When comparing vaccination costs to the losses incurred due to rabies-related fatalities, regardless of herd size, the average cost of vaccinations constituted less than 10% of the estimated economic loss. According to this research, this underlines the efficacy and economic viability of vaccinations as a viable approach for rabies control [9].

From 2017 to 2021, the number of vaccine doses sold in Rondonia (Table 4) relative to the population of cattle and horses (Table 1) was sufficient to immunize approximately 23–34% of the herd. Both the Spearman correlation and GEE analysis indicate that this risk-based vaccination program has been successful in significantly reducing herbivore rabies in Rondonia, although it has not completely eradicated it. Implementing mandatory vaccination for the entire cattle population in Rondonia (which stood at 16 million in 2021) to address the remaining 5–10 annual rabies cases would pose a substantial financial burden. However, as suggested by the analysis in [9], expanding either mandatory or recommended vaccination to all properties in Rondonia with a history of herbivore rabies is likely to be a cost-effective approach to further diminish rabies occurrences without introducing ecological risks.

Additionally, our data demonstrate that the incidence of rabies in cattle and horses is positively associated with the rate of printed educational materials. Printed material had no significant spearman correlation, yet had an extremely significant GEE estimation. We believe that this discrepancy may be related to the fact that the Spearman correlation test is a non-parametric method, where the specific value of the data is not relevant. Only the position of that value in relation to the positions of the others is considered. This is different from the Pearson correlation and GEE tests (which are significant), where the actual value of the data is considered.

We can hypothesize two distinct scenarios to explain this result: The first would be linked to IDARON’s actions of printing educational materials reactively in response to rabies occurrence in domestic herbivores, with the aim of educating farmers and preventing new cases. The second scenario would involve the effectiveness of distributing the printed materials to farmers leading to the increased reporting of suspected cases and, consequently, case detection and incidence increasing. On the other hand, the implementation of meetings, speeches, courses, and media dissemination (such as television and radio) did not appear to have a relationship with the incidence of rabies. However, we believe that these actions should not be discontinued, as they contribute to raising awareness among the general population (including farmers) about this infectious disease and can potentially contribute to rabies control through the reporting of suspected cases [39]. Promoting awareness and the reporting of suspected cases and detection by testing supports the vaccination and bat treatment activities which were effective. Within cattle owners in Buthan, there was a noticeable absence of in-depth understanding concerning rabies, including vulnerable hosts, pathways of transmission, the impact of rabies infection on human health, and suitable health-related responses. This research underscores the urgency of bolstering rabies education initiatives in rural areas to bridge these informational voids [40]. Similarly, our findings emphasize the ongoing importance of promoting health education in the state of Rondônia, Brazil.

Lastly, we emphasize that because we used data obtained from the IDARON’s database, which was supplied by different managers and professionals over approximately 20 years, there may be some biases present in this database, such as errors in data annotation and an inability to distinguish data = 0 (absence) from data that are unknown, missing, or not recorded (zero values in Table 3, Table 4 and Table 5). When it comes to actions taken by official agencies, such as IDARON, the absence of information implies a value of zero because if any action had taken place, it would have been recorded. In the case of disease cases, we always consider the recorded number because there is no way to determine the real occurrence counts due to the possibility of under-reporting. However, in some other situations, it is possible that certain data may have only started being recorded after a certain period, and they might not have been zero. Assessing this impact on our results is challenging. Nevertheless, given the large span of years of the data included in this study, we believe this impact is likely to be minimal, though it is worth mentioning.

## 5. Conclusions

Ultimately, the large and statistically significant decrease in rabies incidences among cattle and equines can be primarily attributed to the control measures enacted by IDARON. These measures involve the administration of anticoagulant poison to bats and the vaccination of domestic herbivores, while printing educative materials may help to detect rabies cases by IDARON. However, it is crucial to recognize that the success of these interventions is underpinned by a range of additional program activities. These activities encompass diagnostic testing, the registration and monitoring of shelters, and bat capture events. These auxiliary actions play an indispensable role in facilitating the treatment of bats and the execution of the vaccination program.

The existing rabies control activities, while effective, come at a cost and must be continued to maintain disease control. In addition, culling vampires has inherent risks of unintended ecological consequences. Consequently, it remains important for IDARON to continue to consider the potential of emerging control strategies, such as a transmissible bat rabies vaccine, that may enhance the effectiveness of the rabies control program with less of an ecological impact. As such, a holistic evaluation of these methods and their potential consequences is prudent for the ongoing success of the initiative.

## Figures and Tables

**Table 1:** Number of rabies cases and incidence risk (cases per one million animals) affecting domestic herbivores in the state of Rondônia, Brazil, from 2002 to 2021.

Year	Bovine				Equines				Bovine and Equines			
	Cases	Population	Incidence	95% C.I.	Cases	Population	Incidence	95% C.I.	Cases	Population	Incidence *	95% C.I.
2002	15	8,606,388	1.74	1.06−2.88	4	164,606	24.30	9.45−62.49	19	8,770,994	2.17	1.39−3.38
2003	32	9,622,179	3.33	2.36−4.69	3	167,487	17.91	6.09−52.67	35	9,789,666	3.58	2.57−4.97
2004	22	10,677,040	2.06	1.36−3.12	3	177,582	16.89	5.75−49.67	25	10,854,622	2.30	1.56−3.40
2005	7	11,350,085	0.62	0.30−1.27	1	177,852	5.62	0.99−31.85	8	11,527,937	0.69	0.35−1.37
2006	11	11,478,086	0.96	0.54−1.72	0	167,640	0.00	0.00−22.91	11	11,645,726	0.94	0.53−1.69
2007	25	11,012,991	2.27	1.54−3.35	3	188,357	15.93	5.42−46.83	28	11,201,348	2.50	1.73−3.61
2008	18	11,176,201	1.61	1.02−2.55	2	188,838	10.59	2.90−38.62	20	11,365,039	1.76	1.14−2.72
2009	16	11,526,439	1.39	0.85−2.56	0	197,067	0.00	0.00−19.49	16	11,723,506	1.36	0.84−2.22
2010	8	11,841,496	0.68	0.34−1.33	0	161,050	0.00	0.00−23.85	8	12,002,546	0.67	0.34−1.32
2011	1	12,068,525	0.08	0.01−0.47	0	170,019	0.00	0.00−22.59	1	12,238,544	0.08	0.01−0.46
2012	2	12,212,647	0.16	0.04−0.60	0	210,148	0.00	0.00−18.28	2	12,422,795	0.16	0.04−0.59
2013	8	12,280,946	0.65	0.33−1.29	1	207,287	4.82	0.85−27.33	9	12,488,233	0.72	0.38−1.37
2014	4	12,744,340	0.31	0.12−0.81	1	166,507	6.01	1.06−34.02	5	12,910,847	0.39	0.17−0.91
2015	2	13,391,818	0.15	0.05−0.54	1	165,130	6.06	1.07−34.31	3	13,556,948	0.22	0.08−0.65
2016	1	13,682,200	0.07	0.01−0.41	1	164,607	6.08	1.07−34.41	2	13,846,807	0.14	0.04−0.53
2017	3	14,091,378	0.21	0.07−0.63	0	166,722	0.00	0.00−23.04	3	14,258,100	0.21	0.07−0.62
2018	4	14,337,157	0.28	0.11−0.72	0	192,463	0.00	0.00−19.96	4	14,529,620	0.28	0.11−0.71
2019	5	14,349,219	0.35	0.15−0.82	3	202,631	14.81	5.04−43.53	8	14,551,850	0.55	0.28−1.08
2020	9	14,804,398	0.61	0.32−1.16	0	192,463	0.00	0.00−19.96	9	14,996,861	0.60	0.32−1.14
2021	8	16,234,295	0.49	0.25−0.97	0	201,055	0.00	0.00−19.11	8	16,435,350	0.49	0.25−0.96
Total	201	12,374,391	16.24	14.15−18.65	23	181,476	126.74	84.46−190.18	224	12,555,867	17.84 **	15.65−20.33

* Cases per one million animals; ** Calculated by the relation among the sum of cases and the mean population size in the period.

**Table 2:** Results of the long-term trend analysis of the rabies incidence rate in cattle, equines, and the overall number of rabies cases in the state of Rondônia. The analysis covers the period from 2002 to 2021 and includes the rate of variation along with its respective 95% confidence interval.

Rabies Occurrence	Beta 1 Coefficients	Annual Percentage Variation (95% CI)	*p*-Value
Incidence in bovines	−0.10703	−21.84 (−39.26–0.57)	0.0548
Incidence in equids	−0.6239	−76.23 (−96.65–68.73)	0.1141
Number total of cases	−0.07988	−16.80 (−32.81–3.02)	0.0873
Risk of positives per 10,000 farms	−0.09806	−20.21 (−35.39–1.46)	0.0374

**Table 3:** Raw data related to bat shelters, including monitoring, registering, capture events, number of captured and treated bats, and number of bats sent to laboratory for rabies diagnosis.

Year	Annual Numbers					
	Monitored Shelters	Registered Shelters	Capture Events	Captured Bats	Treated Bats (%)	Bats Sent to Laboratory
2002	0	0	0	0	0 (-)	0
2003	0	1	2	2	2 (100)	0
2004	0	18	19	27	26 (96)	0
2005	0	9	17	25	24 (96)	1
2006	0	29	40	46	40 (87)	4
2007	0	25	33	58	39 (67)	4
2008	0	2	23	23	22 (96)	0
2009	0	0	0	0	0 (-)	0
2010	13	8	116	130	115 (88)	4
2011	29	14	127	181	166 (92)	9
2012	45	27	128	93	87 (94)	2
2013	60	21	170	97	95 (98)	1
2014	18	6	96	105	97 (92)	5
2015	22	8	84	68	62 (91)	6
2016	17	8	58	49	48 (98)	1
2017	6	6	33	102	101 (99)	1
2018	2	2	9	5	4 (80)	0
2019	0	0	19	12	10 (83)	0
2020	5	5	12	10	9 (90)	1
2021	4	1	48	71	47 (66)	1
Total	221	190	1034	1104	994 (90)	40
min	0	0	0	0	0 (66)	0
max	60	29	170	181	166 (100)	9
mean	11.05	9.5	51.7	55.2	49.7 (90)	2
median	3	7	33	47.5	39.5 (92)	1

**Table 4:** Raw data related to selling vaccines and anticoagulant poison.

Year	Annual Numbers		
	Doses of Vaccines Sold	Number of Anticoagulant Poison Units Sold	Number of Farms That Acquired Anticoagulant Poison or Vaccine
2002	0	0	0
2003	895,325	0	0
2004	1,453,130	0	0
2005	2,262,340	140	559
2006	2,022,685	395	1312
2007	2,979,883	681	4677
2008	4,971,232	895	17,169
2009	3,804,382	708	23,269
2010	3,078,863	907	8481
2011	3,026,684	530	9527
2012	2,386,370	572	10,835
2013	3,558,702	356	17,780
2014	3,385,230	483	18,015
2015	6,117,720	482	0
2016	5,241,495	352	0
2017	4,800,613	246	0
2018	3,365,151	282	0
2019	3,551,717	698	0
2020	4,083,343	1434	116
2021	4,813,093	1200	395
Total	65,797,958	10,361	112,135
min	0	0	0
max	6,117,720	1434	23,269
mean	3,289,898	518.05	5606.75
median	3,375,191	482.5	477

**Table 5:** Raw data related to educational initiatives (meetings, courses, and lectures; media; and units of printed materials) and diagnostic tests carried out.

Year	Annual Numbers			
	Meetings, Courses, and Lectures	Media	Printed Material	Diagnostic Tests
2002	0	0	0	32
2003	6	0	153,000	81
2004	1	0	0	86
2005	728	1045	71,205	89
2006	608	96	53,963	49
2007	767	145	102,601	75
2008	820	234	88,760	114
2009	439	68	59,208	106
2010	456	223	43,674	100
2011	670	51	32,938	106
2012	442	151	34,417	118
2013	306	76	43,279	136
2014	168	138	24,746	126
2015	353	7	14,218	101
2016	186	10	8295	84
2017	105	3	2432	67
2018	59	1	3236	63
2019	81	3	4696	55
2020	13	2	82	74
2021	6	0	117	51
Total	6214	2253	740,867	1713
min	0	0	0	32
max	820	1045	153,000	136
mean	310.7	112.65	37,043.35	85.65
median	246	30.5	28,842	85

**Table 6:** Summary statistics of outcome and explanatory variables used in study, including minimum value, first quartile, median, mean, third quartile, and maximum value.

Variable	Min.	1st Qu.	Median	Mean	3rd Qu.	Max
Incidence risk for bovines and equines	0.08	0.26	0.63	0.99	1.46	3.58
Ratio of monitored shelters	0.00	0.00	0.29	1.21	1.80	6.63
Ratio of registered shelters	0.00	1.94	7.36	10.64	17.42	31.59
Ratio of capture events	0.00	1.72	3.73	5.70	9.24	18.79
Ratio of captured bats	0.00	1.19	5.03	6.10	10.50	20.78
Ratio of treated bats	0.00	1.01	4.37	5.50	9.92	19.06
Ratio of bats sent to laboratory	0.00	0.00	1.06	2.21	4.44	10.33
Ratio of diagnostic tests carried out	4.74	7.00	10.13	9.65	12.28	15.03
Ratio of vaccine doses sold	0.00	26.41	36.18	35.91	44.89	64.67
Ratio of doses of anticoagulant poison sold	0.00	2.93	5.14	5.60	8.13	13.74
Proportion of farms that acquired anticoagulant poison	0.00	0.00	0.50	6.42	11.24	27.68
Ratio of education actions	0.00	0.50	2.65	3.53	5.65	9.83
Ratio of midias	0.00	0.02	0.34	1.28	1.53	11.74
Ratio of educative material	0.00	0.33	3.22	4.39	6.17	20.46

**Table 7:** Generalized estimating equations multiple model for rabies occurrence in the state of Rondonia, Brazil, 2002 to 2021.

Outcome	Explanatory Variables	Estimate	Standard Error	*p*-Value
Incidence risk for bovines and equines	Ratio of treated bats	−0.06223	0.01311	2.1 × 10^−6^
	Ratio of vaccine doses sold	−0.02125	0.0081	0.00898
	Ratio of educative printed material	0.10309	0.02145	1.5 × 10^−6^

## Data Availability

Raw data are available in the manuscript.
